# Pilots’ and cabin crews’ psychosocial work environment in relation to mental health and fitness-to-fly safety behaviors: latent profile analyses

**DOI:** 10.3389/fpsyg.2026.1740908

**Published:** 2026-03-18

**Authors:** Filippa Folke, Kimmo Sorjonen, Marika Melin

**Affiliations:** Division of Psychology, Department of Clinical Neuroscience, Karolinska Institute, Solna, Sweden

**Keywords:** aviation, occupational health, organizational and psychosocial work environment, safety behaviors, self-declaration of unfitness

## Abstract

**Background:**

Increased competition in European aviation has led to cost-cutting measures and restructuring, resulting in more demanding psychosocial work environments for aircrew (pilots and cabin crew). Understanding how different combinations of psychosocial work environment risks relate to mental health and safety behaviors could guide targeted interventions to enhance wellbeing and flight safety.

**Methods:**

This cross-sectional study used self-reported data collected in Europe in 2022 and examined combinations of self-rated psychosocial factors, mental health (i.e., depression, anxiety, and fatigue), and fitness-to-fly safety behaviors (i.e., presenteeism and self-disclosure of unfitness) among pilots (*N* = 4,960) and cabin crew (*N* = 1,684). Latent profile analyses were used to identify psychosocial work environment profiles, followed by statistical comparisons of organizational covariates, mental health, and fitness-to-fly safety behaviors.

**Results:**

Among pilots and cabin crew, four profiles of the psychosocial work environment were identified: *High-Flying*, *Roster Empowered*, *Collegially Supported*, and *Strained*. Profiles reflecting less favorable psychosocial work environments reported poorer mental health and safety behaviors. The *Strained* profile displayed a combination of poor mental health and lower willingness to self-disclose unfitness to the employer, suggesting a mental health non-disclosure pattern that could reinforce health and safety risks. Offering a minimum guaranteed pay, increasing perceptions of roster influence, and strengthening collegial support emerged as potential organizational and psychosocial intervention areas warranting further attention.

**Conclusion:**

By focusing on health and safety in relation to the psychosocial work environment, we show that addressing psychosocial risks could be associated with dual benefits, enhancing occupational health, while simultaneously improving flight safety.

**Practical application:**

The study offers practical insights into which organizational and psychosocial factors could be targeted to improve mental health and safety behaviors among aircrew in Europe.

## 1 Introduction

In aviation, the wellbeing of pilots and cabin crew is important not only for individual health but also for flight safety. Although accidents are often attributed to systemic failures, the human factor - including the mental and physical fitness of operators—plays an important role in ensuring safety. Aircrew, i.e., pilots and cabin crew, are therefore legally required to refrain from duty when their mental or physical state may endanger flight safety (European Commission, 2008, 2011, European Commission Regulation, No 216/2008, No 1178/2011). Nevertheless, accident statistics reveal that aircrew do not always comply with these regulations, as aviation accidents continue to be attributed to crew unfitness (Kelly et al., 2011; Shappell et al., 2007; Zhu et al., 2024).

Mental health impairments, such as fatigue, depression, and anxiety, are an increasing concern among aircrew (e.g., Cahill et al., 2023; Wen et al., 2023). When such impairments with potential safety implications occur, crew face the decision of self-declaring unfitness to fly or continue working despite being unfit (presenteeism). Unlike many other workplaces where colleagues or managers may recognize mental health problems (Bertilsson et al., 2021), aircrew operate in rotating teams with limited managerial oversight. Self-declaration of unfitness thus represents a central safety behavior for flight safety, while presenteeism reflects non-adherence to safety regulations, increasing safety risks (Johansson and Melin, 2018), and limiting access to support. Consequently, decisions about fitness to fly are also connected to opportunities for help-seeking and early intervention. Although research on these fitness-to-fly safety behaviors (i.e., presenteeism and self-declaration of unfitness) among pilots is growing (e.g., Folke and Melin, 2025; Hoffman et al., 2022), they remain understudied among cabin crew.

In response to these challenges regarding mental health issues, additional regulations and interventions have been implemented to safeguard aircrew mental health and prevent safety-critical escalation (e.g., psychological assessment and peer support programs). Nevertheless, despite such initiatives, barriers to self-declaration of unfitness may persist (Bråstad et al., 2024; Minoretti, 2025).

### 1.1 Aircrew mental health, fitness-to-fly safety behaviors, and psychosocial risks

Despite a legal and operational focus on fitness, mental health issues—such as fatigue, anxiety, and depression—are frequently reported by aircrew (Bendak and Rashid, 2020; Wen et al., 2023; Wu et al., 2016). Among pilots, fatigue prevalence is typically reported at two-thirds or more across different contexts (Jackson and Earl, 2006; Zhang et al., 2022). Estimates from the World Health Organization (WHO) suggest a global prevalence of 4.4% for depression and 3.6% for anxiety in the general population (World Health Organization [WHO], 2017). In pilots, however, prevalence estimates vary widely, with reports ranging from below global figures to substantially higher levels (40%) (Ackland et al., 2022). Nevertheless, concerns of under-reporting persist, given potential negative career implications of self-disclosure of unfitness (Parker et al., 2001; Lollis et al., 2009).

Among cabin crew, fatigue prevalence is likewise reported at high levels (around 60–75%), alongside increased sleep-related difficulties. A scoping review (Wen et al., 2023) suggests elevated levels of depression and fatigue relative to the general population, and in some studies, a higher prevalence than among pilots, although findings are not unanimous (Feijo et al., 2014; McNeely et al., 2018; Omholt et al., 2017). Up to 40% of cabin crew appeared at risk of depression, with around 20% reporting anxiety symptoms (Wen et al., 2023). As with pilots, reporting may be influenced by career constraints, making prevalence estimates uncertain.

Collectively, these findings point to the work context as a relevant domain for understanding mental health impairments among aircrew. Broad occupational-health research demonstrates that psychosocial working conditions are associated with mental health (e.g., Harvey et al., 2017; Niedhammer et al., 2021), and across safety-critical industries, suboptimal work environments can impair mental health and predispose to safety deviations (Derdowski and Mathisen, 2023; Folke and Melin, 2022; Nahrgang et al., 2011; Shappell et al., 2007). Consistent with this, aircrew report several psychosocial risks in their work environment that have potential negative implications for both health and safety (Cahill et al., 2023). European survey data on aircrew following the COVID-19 pandemic showed several challenges for health and safety, including a deteriorating safety climate, work intensification, demanding and irregular schedules with limited employee influence, strained relations with management, and job insecurity (Folke and Melin, 2022; Jorens and Valcke, 2025). The sector has also seen a rise in atypical employment arrangements, which are associated with poorer health and safety outcomes among aircrew (Jorens et al., 2015; Jorens and Valcke, 2025).

The most prevalent risk factors examined for aircrew concerning various aspects of mental health are workload, disruptions of circadian rhythms, long workdays, and pre-duty sleep (Bendak and Rashid, 2020; Marqueze et al., 2023; O’Hagan et al., 2016; Wen et al., 2023). Crew also point to psychosocial aspects, such as schedule regularity (Tsaur et al., 2020), schedule control (Castro et al., 2015), and company support (Van Den Berg et al., 2020) as important for mental health.

In relation to safety behaviors, limited research suggests that pilots report presenteeism at rates similar to the general population (Johansson and Melin, 2018). A poor safety climate has been linked to depressive symptoms, increased safety deviations, and a greater likelihood of presenteeism (Folke and Melin, 2022; Melin et al., 2018). Reported reasons for continuing to fly while unfit include job insecurity, fear of career consequences, poor safety climate, financial pressures, and a sense of loyalty to the airline and colleagues (Folke and Melin, 2022, 2025). Reluctance to disclose health impairments to employers or medical examiners appears driven by similar factors, embedded in a broader system of interrelated constraints, including stigma, job-security and medical-certification concerns, limited trust in confidentiality, regulatory constraints, and organizational barriers (Cross et al., 2024; Hoffman et al., 2022; Minoretti, 2025; Patel et al., 2023; Strand et al., 2022), alongside challenges of accurately self-assessing the condition’s relevance to flight safety (Folke and Melin, 2025; Strand et al., 2022), thereby limiting opportunities for help and support.

Together, these studies show that aircrew experience multiple psychosocial risks in their work environment, with implications for both mental health and barriers to fitness-to-fly safety behaviors. Although psychosocial risks—such as demanding rosters, job insecurity, limited control, and strained management relations—are increasingly documented for aircrew (e.g., Cahill et al., 2023; Folke and Melin, 2024; Jorens and Valcke, 2025), and align with well-established determinants of workplace mental health (Harvey et al., 2017), no studies have examined how these conditions co-occur within the work environment, nor how such combinations relate to both mental health and fitness-to-fly safety behavior. Addressing this gap requires examining how psychosocial working conditions cluster within individuals, rather than studying each factor in isolation.

### 1.2 A person-centered approach to psychosocial risks

Most research on psychosocial work factors relies on variable-centered approaches, examining associations between variables at the group level (Howard and Hoffman, 2018; Ong and Johnson, 2023). While informative, this perspective assumes homogeneity and cannot capture how psychosocial conditions cluster within individuals and relate to outcomes.

This limitation is also evident in the application of the Job Demands-Resources (JD-R) model (Demerouti et al., 2001), which conceptualizes how psychosocial work environment factors influence employee health (Bakker and Demerouti, 2017; Demerouti et al., 2001) and has been broadened in subsequent research to include safety-related behaviors and outcomes (Derdowski and Mathisen, 2023; Nahrgang et al., 2011). In this model, job demands (e.g., work pressure, job insecurity) are associated with mental health through a health-impairment pathway, whereas job resources (e.g., social support, management trust) foster motivation and engagement through a motivational pathway (Bakker and Demerouti, 2017). However, the core interactionist assumption of the model, that resources can buffer the negative impact of demands (Bakker et al., 2005), has received mixed support. While some studies show buffering effects, meta-analyses suggest additive effects explain more variance (Gonzalez-Mulé et al., 2021; Huth and Chung-Yan, 2023). Research on safety behaviors shows similar patterns, suggesting that demands and resources have a direct effect on safety behaviors, with limited effects for buffering (Nahrgang et al., 2011). While studies suggest buffering effects of safety climate, as well as supervisory or collegial support (e.g., Bronkhorst, 2015; Guo et al., 2019; Probst, 2004), others find no such moderating effects (e.g., Sampson et al., 2014). Nevertheless, high demands (e.g., work pressure and job insecurity) and low resources (e.g., low job autonomy and supervisory support), predict safety violations and reduced compliance (Christian et al., 2009; Derdowski and Mathisen, 2023; Nahrgang et al., 2011).

These mixed results, combined with the dominance of variable-centered analyses, have prompted calls for person-centered approaches that capture how demands and resources co-occur in practice (Howard and Hoffman, 2018; Ong and Johnson, 2023). In contrast to variable-centered analyses, person-centered analyses identify subgroups of individuals who share similar psychosocial work environment patterns (Morin et al., 2018; Spurk et al., 2020). This approach allows examination of how these profiles, as the independent variable, relate to outcomes (Kusurkar et al., 2020; Morin et al., 2018), such as health and safety behaviors. Moreover, they can identify factors associated with profile membership, such as type of employment or individual characteristics (e.g., gender). Person-centered analyses therefore complement traditional methods by identifying psychosocial work environment profiles that may require targeted interventions (Kusurkar et al., 2020). In aviation, such profiles may reveal high-risk subgroups of mental health issues and deviations in fitness-to-fly safety behaviors, not detectable through variable-centered methods.

### 1.3 Aims and research questions

This study examines how combinations of psychosocial work environment factors relate to mental health and fitness-to-fly safety behaviors in aviation. Using an exploratory person-centered approach (Meyer and Morin, 2016), we allow psychosocial work environment profiles to emerge empirically rather than specifying hypothetical combinations in advance, reflecting that the difference between job demands and resources is not always clear-cut (Bakker and Demerouti, 2024; Ong and Johnson, 2023). We address three research questions:

**Identify latent profiles based on the psychosocial work environment**
*RQ1*: What profiles of self-rated psychosocial work environments exist among cabin crew and pilots? Can similar profiles be identified among pilots and cabin crew?
**Examine profile membership**
*RQ2*: Do groups of cabin crew or pilots, depending on organizational and individual covariates (such as employment type or gender), tend to belong to a specific profile?
**Latent profile differences in mental health and safety behaviors**
*RQ3*: How do the profiles of self-rated psychosocial work environments vary concerning pilots’ and cabin crews’ mental health and safety behaviors?

Together, these questions enable a shift from identifying risk factors to identifying risk groups—a prerequisite for targeted safety and wellbeing interventions in aviation.

## 2 Materials and methods

### 2.1 Data collection, procedure, and participants

This study employed a cross-sectional design using survey data from two self-report web surveys aimed at cabin crew and pilots (excluding military pilots) in Europe. Given that the surveys were extensive, completion rates were somewhat lower (52% for cabin crew, 63% for pilots), but still within or above the range typically observed for long web-based surveys (Kost and Correa da Rosa, 2018). Completion rate analyses are available in Supplementary Appendix 1. Demographic characteristics of the samples are presented in Table 1.

**TABLE 1:** Organizational and individual demographics of the samples (cabin crew and pilots).

Demographics	Cabin crew (*N* = 1,684)		Pilots (*N* = 4,960)	
	N	n (%)	N	n (%)
Organizational demographics				
Type of service	1,679		4,958
Scheduled passenger service		1,554 (92.6)		4,223 (85.1)
Non-scheduled (charter) passenger service		75 (4.5)		178 (3.6)
Business aviation service		34 (2.0)		50 (1.0)
Freight/cargo		–		343 (6.9)
Other		16 (1.0)		164 (3.3)
Type of operation	1,681		4,959
Intercontinental/long haul		196 (11.7)		1,395 (28.1)
International + regional/short haul		604 (35.9)		2,522 (50.9)
Regional only		88 (5.2)		517 (10.4)
Mixed (long and short haul)		793 (47.2)		439 (8.9)
Other		–		86 (1.7)
Employment type	1,680		4,953
Typical		1,541 (91.7)		4,552 (91.9)
Atypical		139 (8.3)		401 (8.1)
Minimum pay, regardless of flown hours	1,681		4,946
Yes		1,531 (91.1)		4,495 (90.9)
No		150 (8.9)		451 (9.1)
Position	1,615		4,345
Cabin supervisor/purser		532 (32.9)		–
Air host/hostess/cabin crew		1,083 (67.1)		–
Flight captain (FC)		–		2,299 (52.9)
First officer/cruise only pilot (FO)		–		2,046 (47.1)
Individual demographics				
Gender	1,611		4,329
Male		477 (29.6)		4,109 (94.9)
Female		1,126 (69.9)		210 (4.9)
Other		8 (0.5)		10 (0.2)
Relationship status	1,611		4,333
Single		429 (26.6)		426 (9.8)
Married/civil union/living with partner		984 (61.1)		3,538 (81.7)
In a relationship, living apart		198 (12.3)		369 (8.5)

Percentages are based on valid responses. N varies across variables due to differential completion rates. “–” indicates non-applicability for a given role (e.g., pilot vs. cabin crew).

#### 2.1.1 Cabin crew

Data were collected from July to December 2022. Participants were recruited primarily through European central unions, with additional outreach via social media, local unions, and airlines to reach non-unionized crew and those in regions without central union presence. The analytical sample in the current study was 1,684 cabin crew. The mean age of the included cabin crew was 43.6 (SD = 11.05), with an average experience of 17.9 (SD = 10.79) years. The greatest proportion of the sample originated from Northern Europe (37.6%), followed by Southern Europe (31.9%), Western Europe (27.5%), Eastern Europe (2.4%), and other areas (0.7%).

#### 2.1.2 Pilots

Data were collected from April to October 2022. The web survey was primarily spread through e-mail by the European Cockpit Association and their national partners, and also through social media and airlines. The analytical sample consisted of 4,960 pilots. The mean age of included pilots was 43.9 (SD = 9.56), and they had on average been flying for 18.7 (SD = 10.13) years. The greatest proportion of the sample originated from Western Europe (52.4%), followed by Northern Europe (29.3%), Southern Europe (15.9%), Eastern Europe (1.6%), and other areas (0.8%).

#### 2.1.3 Representativeness of samples

The samples are large and diverse, mainly representing mid- to late-career aircrew. Younger and less experienced crew appear underrepresented, partly reflecting data collection in the post-COVID rehiring phase, when airlines prioritized rehiring senior staff. Recruitment through unions may also bias toward more stable employment groups, while Eastern Europe is underrepresented. Completion rates were higher among typically employed and long-haul pilots, while for cabin crew, rates were lower in business aviation compared to other service types. Overall sample sizes are comparable to those reported in previous large European aircrew surveys (Brannigan et al., 2019; Reader et al., 2016; Jorens et al., 2015). The pilot sample corresponds to roughly 6–10% of the estimated pre-pandemic European pilot population (∼50,000–70,000; Reader et al., 2016; Jorens et al., 2015). For cabin crew, the sample represents a smaller fraction of the overall workforce (European Cabin Crew Association [EurECCA], n.d.), though precise figures are not available.

### 2.2 Measures

#### 2.2.1 Latent profiles: psychosocial work environment indicators (RQ1)

Items regarding organizational and psychosocial factors were primarily drawn from well-established instruments, such as the General Questionnaire for Psychological and Social Factors at Work Nordic (QPS Nordic) (Pahkin et al., 2008), the Copenhagen Psychosocial Questionnaire (COPSOQ) (Burr et al., 2019), and the European Work Condition Survey (EWCS) (Parent-Thirion, 2017). Given the extensive scope of the survey, it was necessary to balance survey length with psychometric validity, coverage of areas of interest, and aviation face-validity. Selection and adaptation of items were guided by conceptual relevance to the aviation context and reviewed by pilot and cabin crew reference groups, consisting of union and airline crew representatives, to ensure face validity. All items were on a five-point Likert scale, ranging from *Strongly disagree* (1) to *Strongly agree* (5).

Six indicators were included in this study. Cronbach’s alpha ranged from 0.75 to 0.94, and McDonald’s Omega from 0.76 to 0.94 across samples, indicating acceptable to excellent internal consistency. Detailed descriptions of each indicator, including interpretation and internal consistency (α and ω) are provided in Supplementary Appendix 2, and the full item list is available in Supplementary material.

*1. Roster quality.* Roster quality comprised questions similar to the concept of work time control (Ala-Mursula, 2002) but developed specifically for the target populations in this study. Roster quality comprised five items concerned with roster predictability, stability, and work pace stemming from airline schedules; e.g., “My roster and working days are planned in such a way that I can take necessary breaks during the day (e.g., going to the bathroom, having meals).

*2. Management-employee relations.* Seven items with a focus on staff orientation, management support, management trust and appreciation (Burr et al., 2019; Pahkin et al., 2008; Parent-Thirion, 2017), and psychological contracts (Robinson and Wolfe Morrison, 2000), comprised the Management-Employee Relations variable. An example question was “Management shows interest in the health and wellbeing of the staff.”

*3. Safety climate.* Safety climate was measured using four items from a cross-cultural safety climate instrument previously applied to air traffic management (Reader et al., 2015) and pilots (Reader et al., 2016). The original 19-item questionnaire has been demonstrated to have good psychometric properties and to be a reliable measure, regardless of cultural setting (Reader et al., 2015). The four selected items capture the core dimensions of management commitment to safety, safety communication, safety feedback, and organizational learning from incidents.

*4. Collegial support.* Collegial support was measured using a single item: “If needed, my colleagues support me in my work” (Pahkin et al., 2008).

*5. Roster influence.* Roster influence was measured using a single item: “I feel that I can influence my roster.” The item was created for the study and the target population and is conceptually grounded in the job control and scheduling autonomy dimensions of the psychosocial work environment.

*6. Job insecurity.* Job insecurity was measured using two items, asking respondents whether they worried about losing their job and if they worried about their employment stability (Burr et al., 2019; Heponiemi et al., 2012).

#### 2.2.2 Profile memberships: organizational and individual covariates (RQ2)

##### 2.2.2.1 Organizational covariates

Participants were asked to provide information on their *type of employment*. If participants were directly employed by their airline and had permanent employment, they were referred to as *typically* employed, and if not, *atypically* employed (Jorens et al., 2015). Further, crew were asked if they worked *full-time or part-time*, what *type of service* is offered by their airline, what type of *operation* they fly, and what *position* they held (Brannigan et al., 2019; Jorens et al., 2015; Reader et al., 2016). Participants were further asked about their *nationality*, and whether they had a *guaranteed minimum pay*, regardless of flown hours.

##### 2.2.2.2 Individual covariates

Crew were asked to present information on their *gender*, *age*, *years of work experience*, and *relationship status*.

#### 2.2.3 Latent profile differences: mental health and safety behavior variables (RQ3)

##### 2.2.3.1 Mental health variables

###### 2.2.3.1.1 Depression and anxiety

Depression and anxiety symptoms were assessed using the Hospital Anxiety and Depression Scale (HADS) (Zigmond and Snaith, 1983), comprising 14 items, with seven each for depression and anxiety symptoms. Responses range from no to maximum impairment (0–3 points), with scores ≥ 8 for each subscale indicating mild depression/anxiety. For Depression (α_*cabin*_ = 0.86, ω_*cabin*_ = 0.86, α_*pilots*_ = 0.83, ω_*pilots*_ = 0.83). For anxiety (α_*cabin*_ = 0.85, ω_*cabin*_ = 0.85, α_*pilots*_ = 0.83, ω_*pilots*_ = 0.83).

###### 2.2.3.1.2 Fatigue and sleep issues

Problems with sleep and recovery were measured using four items examining fatigue, recovery, and sleep disturbances (Gustafsson et al., 2008; Linden et al., 2008); e.g., “Do you feel very tired during the working day?” Responses were on a five-point Likert scale, indicating the frequency of problems: (1) low frequency, (5) high frequency (α_*cabin*_ = 0.69, ω_*cabin*_ = 0.70, α_*pilots*_ = 0.75, ω_*pilots*_ = 0.77).

##### 2.2.3.2 Safety behavior variables

###### 2.2.3.2.1 Presenteeism

*Sickness* and *inappropriate presenteeism* were measured using two single-item questions adapted from prior research (Aronsson, 2000; Johns, 2010). Due to the survey’s timing during the post-pandemic ramp-up, the timeframe was adjusted to reflect the current context. The two items were phrased as follows: “During the last six months, or since you returned to work again after the Covid pandemic, have you attended work even though you (were sick/unfit and) should have taken sick leave?” *(Sickness Presenteeism*), and **“**During the last six months, or since you returned to work again after the Covid pandemic, have you attended work despite being unfit for other reasons such as fatigue/mental health/family problems or other issues?” *(Inappropriate Presenteeism).* Responses were categorical (yes/no).

###### 2.2.3.2.2 Self-declaration to employer

One item asked whether respondents would inform their employer if they were feeling depressed or anxious: “If you were feeling depressed or anxious, would you talk to your employer about this?”; yes/no. Although this reflects intention rather than observed behavior, it was treated as an outcome consistent with the Theory of Planned Behavior (TPB) (Ajzen, 1991), which positions intentions as proximal predictors of behavior. This theoretical approach is further supported by research demonstrating the TPB’s applicability to mental health help-seeking among adults (Adams et al., 2022).

### 2.3 Statistical procedure

#### 2.3.1 Identifying latent profiles (RQ1)

Latent profile analysis was employed to identify groups of cabin crew and pilots with similar perceptions of their psychosocial work environment, drawing from suggested best practices (Howard and Hoffman, 2018; Kusurkar et al., 2020; Morin et al., 2018; Spurk et al., 2020). The sample sizes in both groups exceeded recommendations for latent profile analysis, supporting stable profile estimation and classification (Spurk et al., 2020).

First, a confirmatory factor analysis was carried out to validate the structure of the six psychosocial work environment indicators (Table 2). Job insecurity was reversed so higher values on indicators consistently reflected more favorable conditions. Next, deviations from normality were handled by using maximum likelihood estimations and robust standard errors (MLR), and complete cases on the indicators were included in the latent profile analyses, given the person-centered modeling. To assure the exclusion of extreme profiles, outliers were identified using Mahalanobis distance, with a *p*-value of 0.001 used as a cut-off. Multicollinearity was checked for using Pearson’s correlations.

**TABLE 2:** Factor analyses of psychosocial indicators: fit indices for pilots and cabin crew measurement models.

Sample	χ ^2^ (df)	CFI	TLI	RMSEA [90% CI]
Cabin Crew	1,498 (157)	0.928	0.912	0.059 [0.056; 0.061]
Pilots	3,492 (157)	0.953	0.944	0.061 [0.059; 0.063]

χ^2^ (df), Chi-Square (Degrees of Freedom); CFI, Comparative Fit Index; TLI, Tucker-Lewis Index; RMSEA (90% CI), Root Mean Square Error of Approximation (90% Confidence Interval). Model fit was evaluated against conventional cut-offs, CFI/TLI ≥ 0.90 = acceptable, ≥ 0.95 = good, RMSEA ≤ 0.06 (Hu and Bentler, 1999). Neither factor loadings (Δχ^2^ = 143, Δdf = 14, *p* < 0.001) nor intercepts (Δχ^2^ = 993, Δdf = 14, *p* < 0.001) could be assumed to be equal among pilots and cabin crew, i.e., there was no measurement invariance.

A stepwise approach was applied to determine the number of appropriate latent profiles. The fit of different profile solutions was examined using the Akaike information criterion (AIC) and the Bayesian information criterion (BIC). Entropy was examined for group membership confidence. Discrimination of profiles with one fewer class was assessed using the Bootstrapped-Likelihood Ration Test (BLRT) and the Lo-Mendell-Rubin adjusted likelihood ration test (LMR). In line with recommendations, additional profiles containing < 5% of the sample were rejected (Stanley et al., 2017). Finally, profiles were examined based on the ease of conceptual interpretations, theoretical relevance, and added qualitative value (Howard and Hoffman, 2018; Meyer and Morin, 2016). As measurement invariance across pilots and cabin crew could not be established, latent profile analyses were conducted independently within each occupational group. Profile solutions were evaluated within samples and qualitatively across samples to assess structural consistency and potential generalizability (Meyer and Morin, 2016). Similarities across groups are therefore interpreted as convergent patterns rather than as evidence of strict measurement equivalence.

#### 2.3.2 Profile membership and latent profile differences (RQ2 and RQ3)

Profiles were compared based on organizational and individual demographics, and differences in mental health and safety behaviors. Prior to these comparisons, confirmatory factor analyses (CFA) were conducted separately for the mental health variables in the pilot and cabin crew samples to validate their factor structure. Due to missing data and listwise deletion, we examined survey completion rates across profiles.

In latent profile analysis, individuals are assigned to profiles based on their posterior probabilities. As a result, there is inherent classification uncertainty, which, if unaccounted for, can bias comparisons across profiles. To account for this uncertainty, we performed 1,000 bootstrap resampling iterations using each participant’s profile probability. This probability-weighted approach ensures more accurate comparisons, i.e., that differences in the examined variables between the profiles are neither overestimated nor underestimated. While not equivalent to model-based correction methods such as DCAT (Lanza et al., 2013) or BCH in Mplus (Asparouhov and Muthén, 2021), this procedure is in line with the multiple pseudo-class draws method to account for uncertainty in class assignment (Bray et al., 2015) by incorporating individuals’ profile probability distribution into comparisons, rather than assigning them to a single most-likely profile. ANOVAs and chi-square tests were conducted on bootstrapped averages to examine between-profile differences.

**FIGURE 1:**
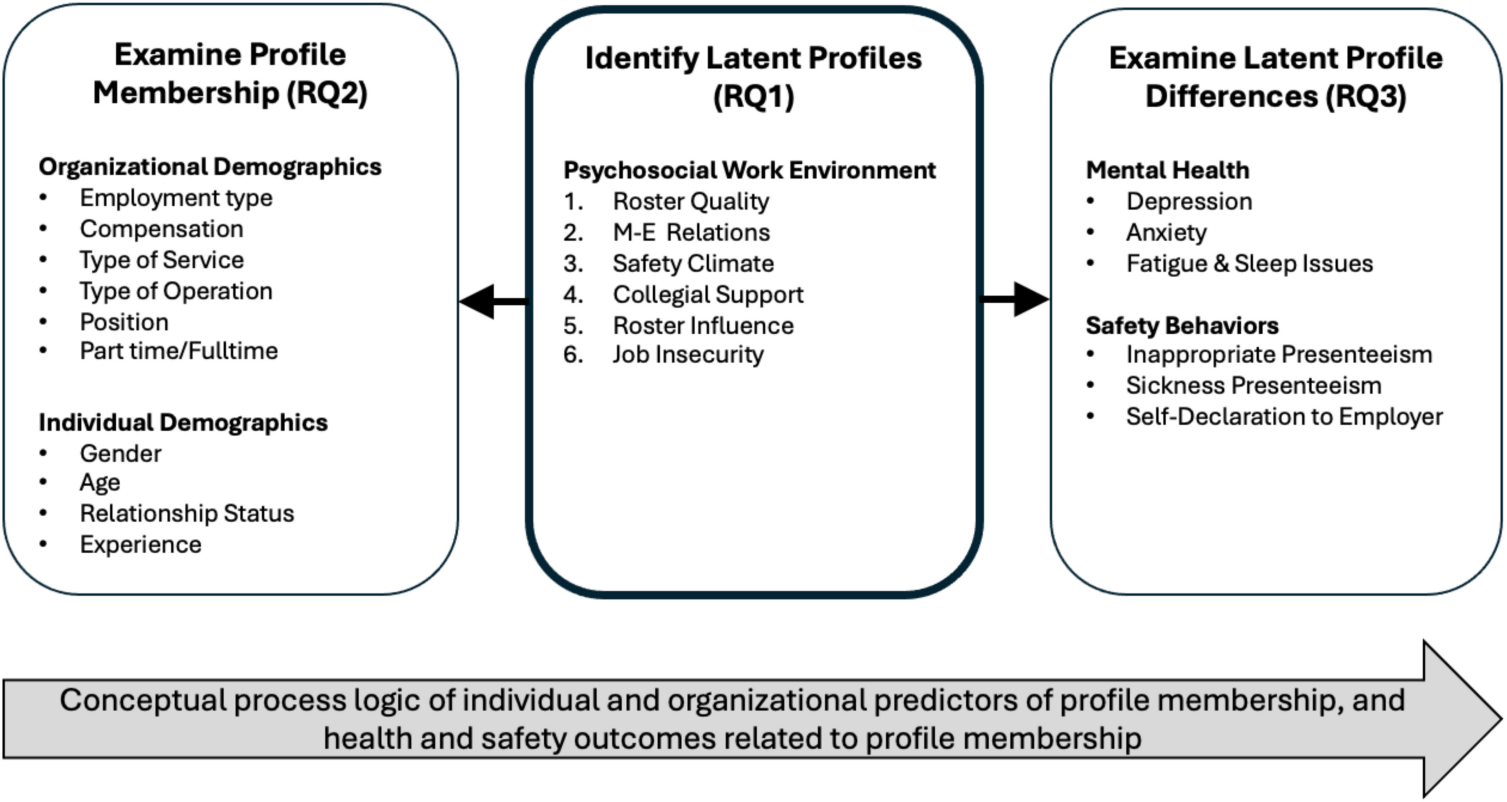
Conceptual process model illustrating the latent profile analysis approach, associated research questions, and variables. The model reflects a process logic in which organizational factors relate to psychosocial profiles, which are associated with health and safety. In line with JD-R theory, six indicators of the psychosocial work environment were used to identify distinct latent profiles (RQ1), capturing different constellations of job demands and job resources. Subsequently, (RQ2) organizational and individual demographics were examined as covariates of profile membership. Finally, (RQ3) profiles were compared on key mental health variables (e.g., depression, anxiety, fatigue) and safety behaviors (e.g., presenteeism, self-disclosure), consistent with the JD-R model’s proposition that the balance between demands and resources influences both mental health and work behavior.

Analyses were performed in R, Jamovi, and SPSS. A conceptual overview of the latent profile analysis approach, associated variables, and research questions can be seen in Figure 1.

## 3 Results

### 3.1 Latent profiles: identifying latent profiles, profile structures and size

In the factor analyses, the proposed six-indicator structure (Figure 1) showed an acceptable fit to data for cabin crew and a good fit for pilots (Table 2) (CFI/TLI ≥ 0.90 = acceptable, ≥ 0.95 = good; Hu and Bentler, 1999). RMSEA values were borderline but still within an acceptable range, especially as the strict cut-off of < 0.06 should not be rigidly used to reject models in large-sample contexts (Marsh et al., 2004). We explored several theoretically plausible model modifications, and among models with comparable fit, we retained the model reported here, as it was most consistent with the theoretical interpretation of the constructs and item wordings. Moreover, reliability estimates were consistently acceptable to excellent across indicators and occupational groups (Supplementary Appendix 2). Due to missing values (cabin crew = 32.8%, pilots = 21.4%) and non-normal distributions, we used listwise deletion and MLR estimation, resulting in 1,699 cabin crew (52.5%) and 5,012 pilots (70.8%). After excluding 15 (0.9%) multivariate outliers in the cabin crew data and 52 (1%) outliers in the pilot data, the final sample in the latent profile analyses was 1,684 cabin crew and 4,960 pilots.

The four-profile solution was selected as the final model in both the pilot and cabin crew samples. This decision was based on a combination of fit statistics, classification accuracy, profile size (Table 3), and a qualitative examination, assuring explanatory value of additional profiles. Although higher-profile solutions showed marginal improvements in fit indices, the four-profile model demonstrated greater substantive meaning and consistency across samples.

**TABLE 3:** Latent profile analyses: model fit statistics for latent profile structures cabin crew (*N* = 1,684) and pilots (*N* = 4,960).

Sample	No. of Profiles	LL	AIC	BIC	SABIC	Entropy	Min-max Prob.	BLRT(p)	LMR(p)	Latent Profile Proportions (%)							
										1	2	3	4	5	6	7	8
**Cabin Crew**	2	−13271.60	26611.25	26795.83	26687.82	0.81	0.80–0.98	0.01	<0.001	83.7	16.3	9.9	**7.4**	7.3	7.0	6.6	6.7
	3	−13157.10	26396.2	26618.79	26488.54	0.82	0.81–0.96	0.01	<0.001	73.7	16.4						
	**4**	**−13055.41**	**26206.83**	**26467.42**	**26314.93**	**0.82**	**0.79-0.94**	**0.01**	**<0.001**	**9.9**	**31.8**	**50.9**					
	5	−12981.35	26072.69	26371.28	26196.56	0.82	0.67–0.95	0.01	<0.001	44.8	10.7	6.6	30.5				
	6	−12940.38	26004.76	26341.35	26144.39	0.78	0.60–0.95	0.01	<0.001	38.1	11.0	6.5	30.2	7.1			
	7	−12952.01	26042.03	26416.62	26197.42	0.76	0.58–0.89	1.00	1.00	37.0	10.5	10.4	25.1	5.8	4.8		
	8	−12940.73	26033.46	26446.06	26204.61	0.68	0.52–0.89	0.02	<0.001	19.0	8.1	11.2	24.3	16.7	8.8	5.2	
**Pilots**	2	−37473.50	75282.27	75503.58	75128.27	0.80	0.92–0.96	0.01	<0.001	61.6	38.4	18.6	**8.6**	12.5	7.4	24.7	6.7
	3	−37376.20	74834.31	75101.19	74970.99	0.73	0.79–0.92	0.01	<0.001	42.8	38.6						
	**4**	**−37309.45**	**74714.98**	**75027.42**	**74874.81**	**0.75**	**0.62–0.93**	**0.01**	**<0.001**	**38.4**	**10.1**	**42.9**					
	5	−36760.85	73631.40	73989.4	73814.94	0.82	0.74–0.94	0.01	<0.001	38.1	6.2	27.0	16.2				
	6	−36611.50	74279.90	74683.47	73553.56	0.71	0.51–0.87	0.01	<0.001	21.2	26.1	18.2	20.0	7.0			
	7	−36524.01	73220.58	73669.71	73415.9	0.74	0.69–0.90	0.01	<0.001	13.8	14.6	15.3	12.7	12.7	6.2		
	8	−36513.79	73176.69	73671.38	73432.78	0.72	0.48–0.90	0.01	<0.001	11.5	17.3	5.0	20.3	14.7	11.8	12.7	

LL, Log Likelihood; AIC, Akaike information criterion; BIC, Bayesian information criterion; SABIC, Sample-size Adjusted Bayesian Information Criterion; BLRT(p), *p*-value for the bootstrapped likelihood ratio test; LMR(p), Lo-Mendell-Rubin adjusted likelihood ratio test. Bolded rows represent the chosen model solutions retained for further analysis.

**FIGURE 2:**
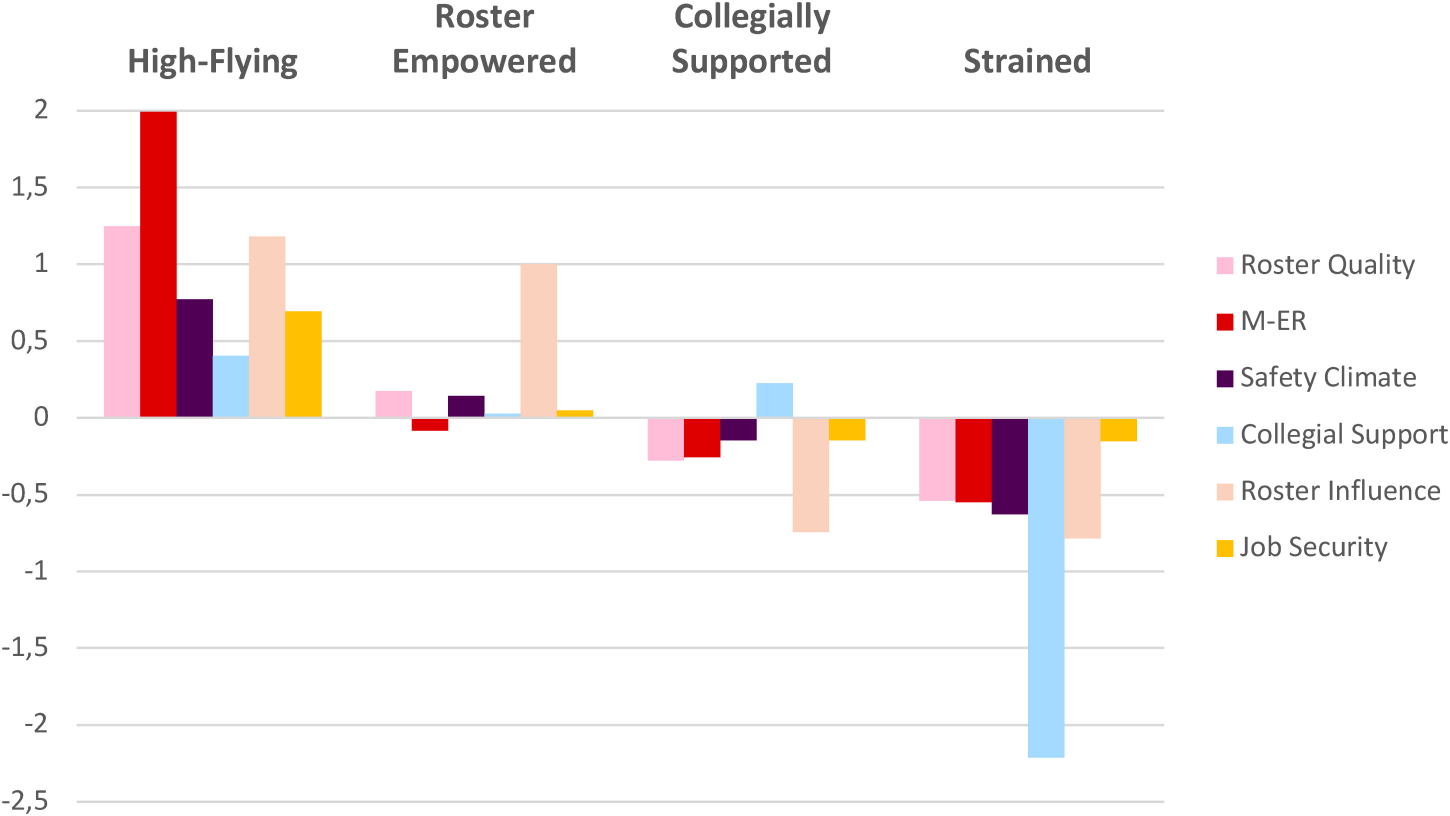
Standardized scores for latent profiles of the psychosocial work environment among cabin crew (*N* = 1,684). M-ER, management-employee relations.

**FIGURE 3:**
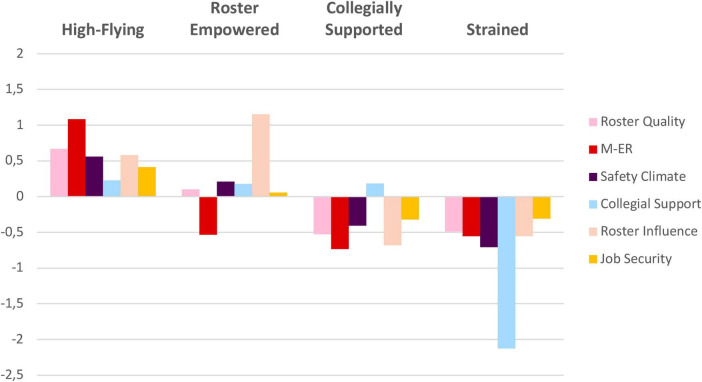
Standardized scores for latent profiles of the psychosocial work environment among pilots (*N* = 4,960). M-ER, management-employee relations.

All psychosocial indicators differed significantly across profiles for both pilots and cabin crew (Supplementary material). Management-employee relations and the perception of roster influence showed the largest between-profile differences (η^2^), followed by collegial support and work-time control, whereas safety climate— and particularly job insecurity—varied least (raw scores, η^2^ values, and *post hoc* results are available in Supplementary material). Because profile structures were similar for pilots and cabin crew, identical profile names were used. Names were informed by the relative standing and graphical pattern of each profile across the six indicators (Figures 2, 3) to concisely reflect their distinguishing characteristics.

Four profiles of the psychosocial work environment were identified: *1. High-Flying Work Environment, 2. Roster Empowered Work Environment, 3. Collegially Supported Work Environment*, and *4. Strained Work Environment* (Figures 2, 3). The *High-Flying* rated their psychosocial work environment highest across all indicators, and the *Strained* displayed below average results on all indicators. The *Collegially Supported* work environment was characterized by below-average experiences across all factors except for collegial support. Meanwhile, the *Roster Empowered* work environment was characterized by generally average levels, with lower ratings in management-employee relations, but higher ratings on roster influence. In both samples, the *Collegially Supported* profile was the most prevalent (cabin crew: 50.9%, pilots: 42.9%) and the *Strained* the least prevalent profile across the samples (cabin crew: 7.4%, pilots: 8.6%) (Tables 4, 5).

**TABLE 4:** Cabin crew profile membership: organizational and individual demographics across profiles (*N* = 1,684).

Covariates							Bootstrap results*^d^*	
	High-flying	Roster empowered	Collegially supported	Strained	Significance (F/*X*^2^)	Effect Size (V/η ^2^)	p (mean, max)	*p* < 0.05
Profile size (%/n)	9.9 (167)	31.8 (535)	50.9 (857)	7.4 (125)				
Organizational demographics								
**Operation**					55.7(9)***	0.11	0.006, 0.221	97.8%
Long haul	10.2	10.5	11.9	16.8	4.30(3)			
Short haul	38.6_b_	28.8_a_	38.3_b_	46.4_b_	20.23(3)***			
Regional only	10.2_c_	2.8_a_	5.5_b_	7.2_bc_	15.8(3)**			
Mixed	41.0_ab_	57.9_c_	44.3_b_	29.6_a_	45.4(3)***			
**Service**					15.87(9)		0.447, 0.994	2.4%
Scheduled services	86.8	93.8	92.6	94.4				
Charter	10.2	3.6	4.2	2.4				
Business	1.8	1.9	2.1	2.4				
Other	1.2	0.8	1.1	0.8				
Atypical vs. typical employment	13.2_b_	5.8_a_	8.4_ab_	11.2_b_	11.06(3)*	0.08	0.451, 0.999	5.4%
Purser vs. cabin crew	38.3_b_	28.0_a_	33.7_b_	41.3_b_	11.69(3)**	0.09	0.01, 0.189	96.6%
No min. pay vs. min. pay	4.2_a_	6.2_a_	10.2_b_	18.4_c_	25.01(3)***	0.12	0, 0.17	100%
Part-time vs. full-time work	27.9_ab_	34.6_b_	21.1_a_	20.0_a_	33.54(3)***	0.14	0.059, 0.768	67.5%
Individual demographics								
Female vs. Others^b^	70.6	68.2	70.7	70.5	1.05(3)		0.079, 0.907	57.9%
Single vs. Others^b^	28.1	27.6	25.3	29.5	1.63(3)		0.453, 0.973	0.8%
Age (years)	43.16_a_	43.17_a_	44.22_a_	41.50_a_	2.67(3)*	0.01	0.017, 0.311	91.3%
Experience (years)	16.52_a_	17.84_a_	18.55_a_	16.11_a_	2.95(3)*	0.01	0.023, 0.335	86.1%
*Post hoc* analysis								
Pre-COVID hire vs. post-COVID hire	80.2_a_	94.2_b_	92.6_b_	96.0_b_	38.78(3)***	0.15	0.106, 0.812	45.6%

Asterisks represent significant differences derived from ANOVA (F) or Chi^2^-test (*X*^2^), *p < 0.05, **p < 0.01, ***p < 0.001, V, Cramer’s V; η^2^, eta squared. ^b^Recoded into binary variable. ^d^Bootstrap results show mean and max p-value across the 1,000 iterations as well as the iteration proportion of *p* < 0.05. Subscripts denote significant pairwise differences (*p* < 0.05). Subscripts (_a, b, c, d_) denote significant pairwise differences (*p* < 0.05).

**TABLE 5:** Pilot profile membership: organizational and individual demographics across profiles (*N* = 4,960).

Covariates							Bootstrap results*^d^*	
	High-flying	Roster empowered	Collegially supported	Strained	Significance (F/*X^2^*)	Effect size (V/η ^2^)	p (mean, max)	*p* < 0.05
Profile size (%/n)	38.4 (1907)	10.1 (499)	42.9 (2127)	8.6 (427)				
Organizational demographics								
**Operation**					151.97(12)***	0.10	0, 0	100%
Long haul	29.2_b_	32.5_b_	26.0_a_	28.8_ab_	10.74(3)*			
Short haul	43.8_a_	50.0_b_	57.1_c_	52.2_bc_	70.96(3)***			
Regional only	15.3_b_	7.0_a_	7.4_a_	8.0_a_	77.76(3)***			
Mixed	8.5	9.6	8.7	10.1	1.50(3)			
Other	3.2_b_	0.8_a_	0.8_a_	0.9_a_	39.04(3)***			
**Service**					57.27(12)***	0.11	0, 0	100%
Scheduled services	84.4	87.9	85.8	82.4	6.93(3)			
Charter	4.2	1.6	3.1	5.4	0.07(3)			
Business	1.3	0.8	0.7	1.4	0.41(3)			
Cargo	5.10	7.0	8.3	8.0	1.42(3)			
Other	4.9	2.6	2.2	2.8	0.48(3)			
Atypical vs. typical employment	4.9_a_	6.0_a_	10.1_b_	14.8_c_	66.69(3)***	0.12	0, 0	100%
Captain vs. first officer	54.9_c_	41.3_a_	51.5_b_	64.6_d_	48.49(3)***	0.11	0, 0	100%
No min. pay vs. min. pay	3.2_a_	6.7_b_	7.6_b_	15.0_c_	90.88(3)***	0.14	0, 0	100%
Part-time vs. full-time work	26.2_a_	35.7_b_	32.2_b_	30.5_ab_	25.17(3)***	0.07	0, 0	100%
Individual demographics								
Female vs. others^b^	5.3	5.4	4.8	2.7	4.95 (3)		0.285, 0.942	7.1%
Single vs. others^b^	10.5	8.8	9.0	12.2	5.42 (3)		0.262, 0.900	3.8%
Age	44.11_b_	41.18_a_	44.19_b_	44.98_b_	14.34(3)***	0.01	0, 0	100%
Experience (years)	18.82_b_	16.22_a_	19.06_b_	19.73_b_	10.98(3)***	0.01	0, 0	100%
*Post hoc* analysis								
Pre-COVID hire vs. post-COVID hire	97.0	96.0	97.7	97.7	5.78(3)		0.538, 0.997	0

Asterisks represent significant differences derived from ANOVA (F) or Chi^2^-test (*X*^2^), *p < 0.05, **p < 0.01, ***p < 0.001, V, Cramer’s V, η^2^, eta squared. ^b^Recoded into binary variable. ^d^Bootstrap results show mean and max p-value across the 1,000 iterations as well as the iteration proportion of *p* < 0.05. Subscripts (_a, b, c, d_) denote significant pairwise differences (*p* < 0.05).

### 3.2 Profile membership: organizational and individual demographics by profile

Tables 4, 5 show profile proportions of cabin crew and pilots by organizational and individual demographics. For pilots, those atypically employed had a greater probability of belonging to the *Strained* (14.8%), than the *High-Flying* profile (4.9%). Among cabin crew, where the pattern was less distinct, the highest proportion of atypically employed individuals was found in the *High-Flying* profile (13.2%), followed by the *Strained* profile (11.2%), and was lowest in the *Roster Empowered* profile (5.8%).

Not having a minimum guaranteed pay regardless of flown hours appeared to be a risk factor for belonging to the *Strained* profile, with 15% of pilots and 18.4% of cabin crew in this profile lacking minimum guaranteed pay—significantly higher than among other profiles (e.g., 4.2% of cabin crew and 3.2% of pilots in the *High-Flying* profile lacked minimum guaranteed pay). Among pilots, the *High-Flying* profile was associated with a higher proportion of individuals flying regionally compared with the other profiles. No significant differences for gender or relationship status across profiles was observed.

#### 3.2.1 *Post hoc* exploratory analyses

Due to discrepancies between the pilot and cabin crew samples in the distribution of atypically employed individuals, a *post hoc* analysis was conducted to explore whether the time of hire (pre-/post-pandemic) differed across profiles. This variable was not included in the initial set of covariates but was collected in the survey and analyzed *post hoc*. While overall experience was inconclusive as a predictor for profile membership, among cabin crew, 19.8% of individuals in the *High-Flying* profile had been hired post-pandemic, compared to 4.0–7.4% in other profiles (Table 4), indicating a significant association. No such differences were observed for pilots (Table 5).

### 3.3 Latent profile differences in mental health and safety behaviors

The factor analyses on the mental health variables showed good fit to the data (Table 6). No difference in completion rate based on profile membership was found. Dropouts from indicators in the LPA to completion of the survey was 12.7% for pilots and 4.3% for cabin crew.

**TABLE 6:** Factor analyses of mental health variables: fit indices for pilots and cabin crew measurement models.

Sample	χ ^2^ (df)	CFI	TLI	RMSEA [90% CI]
cabin crew	721 (132)	0.949	0.940	0.051 [0.048; 0.055]
Pilots	1,701 (132)	0.946	0.937	0.051 [0.049; 0.054]

χ^2^ (df), Chi-Square (Degrees of Freedom); CFI, Comparative Fit Index; TLI, Tucker-Lewis Index; RMSEA (90% CI), Root Mean Square Error of Approximation (90% Confidence Interval).

The proportion of crew above the thresholds for depressive and anxiety symptoms differed across profiles. The *Strained* profile showed the highest levels: 50.4% of cabin crew and 36.7% of pilots reported at least mild depressive symptoms, and 65.3% of cabin crew and 40.5% of pilots reported anxiety symptoms above the threshold. In the most prevalent *Collegially Supported* profile, 34.7% of cabin crew and 24.6% of pilots reported depressive symptoms, and 53.8 and 30.5%, respectively, reported symptoms of anxiety. By contrast, the *High-Flying* profile showed the lowest levels, with 80–95% reporting no symptoms. Full distributions across HADS-A and HADS-D categories (none, mild, moderate, severe) are provided in Supplementary Appendix 3.

**FIGURE 4:**
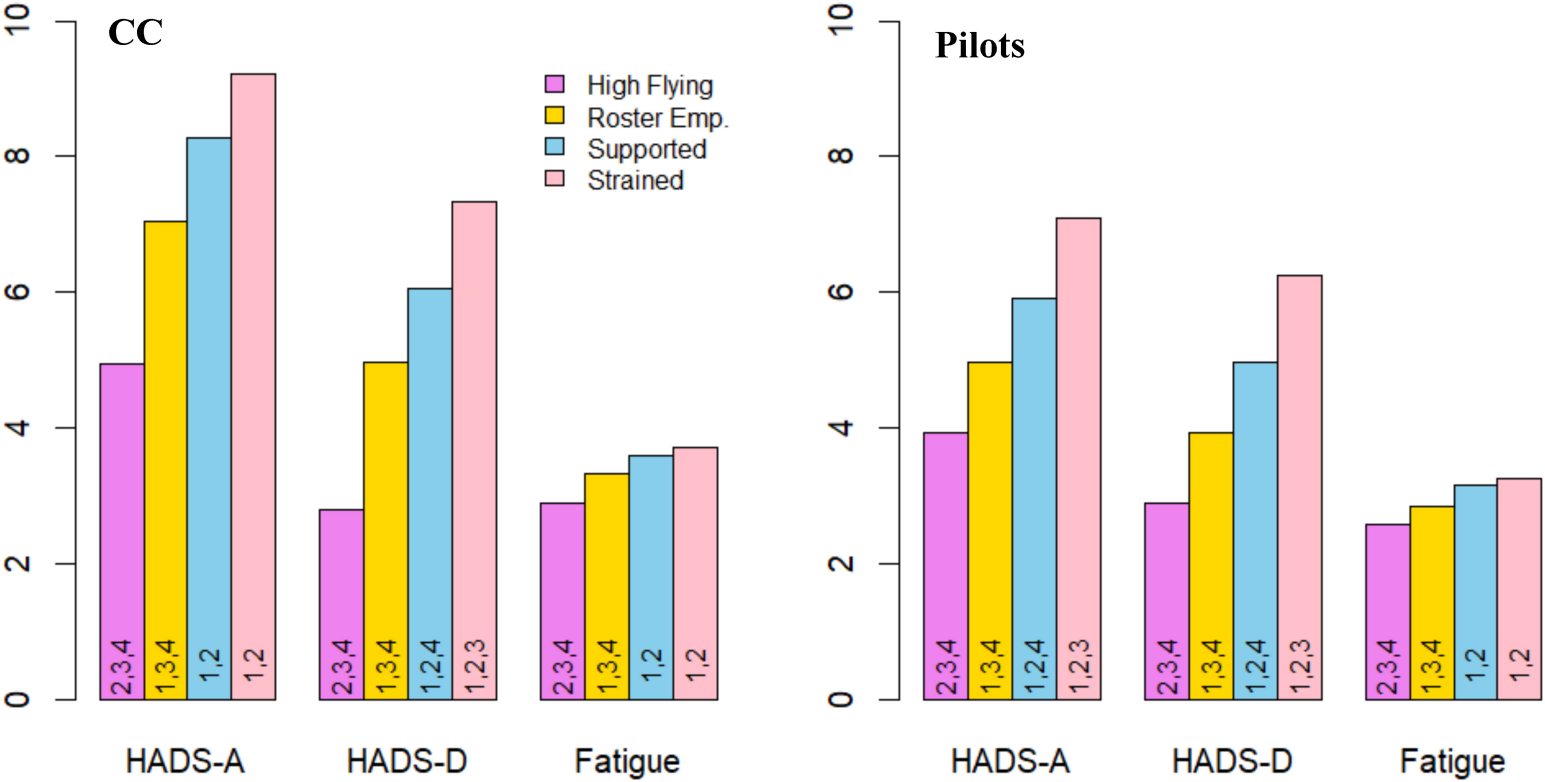
Average scores on mental health variables across profiles for cabin crew and pilots. Average score on HADS-A and HADS-D (min: 0, max: 21), and Sleep and Fatigue Issues (min: 0, max: 5) across profiles. Numbers in bars represent statistically significant differences for that profile on the variable. CC, Cabin crew; HADS-A, Symptom-levels of Anxiety; HADS-D, Symptom-levels of Depression; Fatigue, Sleep and Fatigue Issues; Roster Emp., Roster Empowered; Supported, Collegially Supported. 1 = High-Flying, 2 = Roster Empowered, 3 = Collegially Supported, 4 = Strained.

**FIGURE 5:**
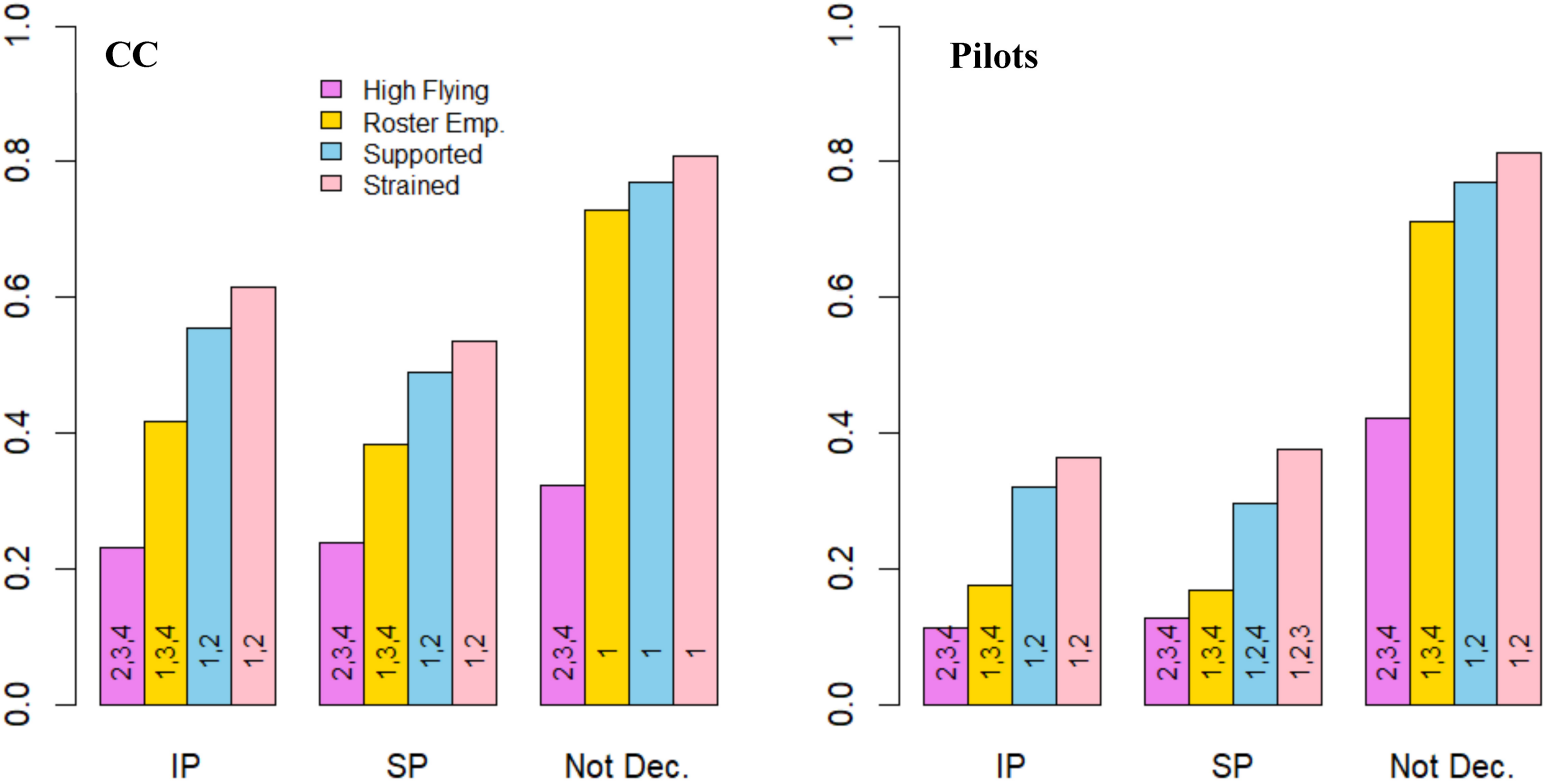
Proportions of fitness-to-fly safety behaviors across psychosocial work environment profiles for cabin crew and pilots. Results are displayed as proportions of the sample. Numbers in bars represent statistically significant differences for that profile on the item. CC, Cabin crew; IP, Inappropriate Presenteeism; SP, Sickness Presenteeism; Not Dec., Would not declare anxiety or depressive symptoms to the employer; Roster Emp., Roster Empowered, Supported, Collegially Supported. 1, High-Flying; 2, Roster Empowered; 3. Collegially Supported; 4, Strained.

For statistical analyses on profile differences in mental health and safety (Table 7), the total scores on HADS-A and HADS-D were used, rather than categories corresponding to symptom levels. Results from *post hoc* tests on differences in mental health and the fitness-to-fly safety behaviors can be seen in raw scores in Figures 4, 5. There were significant differences between most profiles, displaying moderate to large effect sizes for mental health variables and small to moderate effect sizes for safety behaviors (Table 7). Overall, these findings indicate that different psychosocial work environment profiles are associated with variation in self-reported mental health and safety behaviors.

**TABLE 7:** Validation of profiles: latent profile differences in mental health and safety behaviors.

Variables	Cabin crew			Pilots		
	N	F/*X*^2^(df)	η ^2^/Cram. V	N	F/*X*^2^(df)	η ^2^/Cram. V
Health-related variables						
Anxiety symptoms	1,670	38.94(3)***^a^	0.066	4,336	140.7(3)***^a^	0.089
Depressive symptoms	1,673	47.38(3)***^a^	0.078	4,354	163.7(3)***^a^	0.101
Fatigue and sleep issues	1,679	66.72(3)***^a^	0.107	4,484	226.67(3)***^a^	0.132
Safety-related variables						
Sickness presenteeism	1,684	48.02(3)***^a^	0.169	4,441	202.51(3)***^a^	0.214
Inappropriate presenteeism	1,681	77.06(3)***^a^	0.214	4,446	263.04(3)***^a^	0.243
Attitude to self-declaration	1,681	145.14(3)***^a^	0.294	4,931	603.97(3)***^a^	0.350

*p < 0.05, **p < 0.01, ***p < 0.001, ^a^*p* < 0.05 in all 1,000 bootstraps. Differences in mental health-related variables are examined using ANOVA. Frequency differences in safety-behavior variables are examined using *X*^2^.

#### 3.3.1 Sensitivity analyses

As items on presenteeism were retrospective (6months), opening for bias due to differential exposure-related opportunities for attendance and presenteeism, sensitivity analyses were carried out, excluded crew back at work < 6 months, and those in part-time roles. In the cabin crew data, the difference in sickness presenteeism between the *Roster Empowered* and *Strained* groups was no longer statistically significant when cabin crew with back at work < 6 months were excluded. For pilots, excluding part-time workers revealed two clusters in sickness presenteeism, with significant differences between the *High-Flying/Roster Empowered* and the *Collegially Supported/Strained* profiles. Apart from these deviations, overall results remained highly consistent.

## 4 Discussion

In this paper, we aimed to identify combinations of psychosocial work environment factors among pilots and cabin crew and assess profile differences regarding organizational and individual covariates, as well as mental health and fitness-to-fly safety behaviors (i.e., self-declaration of unfitness to employer and presenteeism). Four distinct profiles of self-rated psychosocial work environments were identified among both pilots and cabin crew: *High-Flying* work environment*, Roster Empowered* work environment, *Collegially Supported* work environment, and *Strained* work environment. Profiles reflecting less favorable psychosocial work environments were associated with poorer mental health and fitness-to-fly safety behaviors, and vice versa, reflecting both risk and protective profiles in relation to these factors. Organizational covariates also varied significantly between profiles, emphasizing differences in pay structures and operational contexts. These findings have important implications with regard to targeting psychosocial work environments to support employee mental health and safety behaviors in the aviation industry.

### 4.1 Do certain groups of aircrew tend to belong to a specific profile?

The type of service provided by the airline (e.g., scheduled service, charter, cargo, or business) did not differ significantly across profiles. However, differences emerged in type of operation (long/short haul, regional, mixed). Among pilots, regional flying was more common in the *High-Flyin*g profile, whereas among cabin crew, mixed operations were more prevalent in the *Roster Empowered* profile compared with other profiles. Further, the findings suggest that organizational working conditions, such as the lack of guaranteed minimum pay, may be more prevalent among employees who experience their psychosocial work environment as strained. Economic uncertainty could exacerbate stressors, increase vulnerability to mental health issues, and influence safety-related behaviors such as presenteeism and self-declaration of unfitness.

Atypical employment arrangements, as one example of such organizational practices, have also been associated with poorer mental health (Irvine and Rose, 2024) and reduced safety compliance (Probst et al., 2018), often explained through the concept of job insecurity (Probst, 2002; Probst and Brubaker, 2001; Virtanen et al., 2005). In this study, perceived job insecurity was relatively consistent across three of the four psychosocial work environment profiles but notably lower in the *High-Flying* profile. While the *High-Flying* profile primarily consisted of typically employed pilots, it also included the highest proportion of atypically employed cabin crew. This suggests that perceived psychosocial work experiences do not always align with employment type, particularly for cabin crew. For instance, atypical contracts may be perceived as less insecure when paired with other positive factors, such as good management-employee relations, roster quality, or influence.

Another plausible explanation is that the *High-Flying* profile included more cabin crew hired post-pandemic, a pattern not observed among pilots, raising questions regarding whether transitions between profiles occur as employees gain experience. Favorable self-ratings might be influenced by the initial job satisfaction, and poorer ratings accumulated over time (Boswell et al., 2005, 2009). Although age and industry experience emerged as significant covariates, their effects were mixed and offered limited practical insight. While first-year satisfaction is reported (e.g., Boswell et al., 2009), our measure of experience (in years) may not have been sufficiently sensitive to capture this. Longitudinal research would therefore be needed to examine how psychosocial work profiles evolve over time, or how individuals may transition between them.

Part-time work tended to be more common in the *Roster Empowered* profile, indicating that more off-days due to part-time work, and perhaps a greater opportunity for self-swapping could account for enhanced feelings of roster influence (Kelliher and Anderson, 2010). Supervisory roles were linked to a higher likelihood of belonging to the *Strained* profile, suggesting these positions may carry greater psychosocial risk in aviation.

### 4.2 How do profiles of the psychosocial work environment vary concerning health and safety behaviors?

Our results align with previous research and JD-R theory, showing that high job demands are associated with poorer health and safety compliance, whereas working in a more favorable psychosocial environment is related to better health and enhanced compliance (Bakker and Demerouti, 2017; Christian et al., 2009; Nahrgang et al., 2011). Across both occupational groups, the *Strained* profile, and to a lesser extent the *Collegially Supported* profile, emerged as risk groups. Among cabin crew, 58.3% belonged to these profiles, compared with 51.5% of pilots, indicating that a substantial proportion of European aircrew operate under psychosocial conditions linked to poorer mental health, higher presenteeism, and reduced willingness to self-declare to their employer.

Levels of depressive (4.8–50.4%) and anxiety (12.3–65.3%) symptoms varied markedly across psychosocial work environment profiles. Among individuals classified into the *Strained* work environment profile, reported levels of depressive and anxiety symptoms were notably high, exceeding the upper range reported in previous reviews (e.g., Ackland et al., 2022). By contrast, among individuals classified into the *High-Flying* work environment profile, reported depressive and anxiety symptoms fell below general population norms (World Health Organization [WHO], 2017). Because HADS has limited specificity at standard cut-offs (Pettersson et al., 2015), we also applied a stricter threshold (≥ 11, moderate symptoms). This produced the same pattern for cabin crew, while among pilots, the *Roster Empowered* profile also fell within normative levels of depressive and anxiety symptoms.

Willingness to self-disclose mental unfitness was low across all profiles, consistent with research showing that many pilots avoid available support due to fear and distrust (Cross et al., 2024) and suggesting similar patterns among cabin crew. Among individuals classified into the *Strained* work environment profile, reluctance to self-disclose was particularly pronounced, and coincided with higher levels of mental health symptoms and presenteeism. Whereas self-disclosure tends to increase with symptom severity in general workplaces (Hastuti and Timming, 2021), aviation appears to show the opposite pattern: less favorable psychosocial conditions and poorer mental health co-occur with higher presenteeism and lower willingness to disclose to the employer. Together, this suggests a *mental health non-disclosure pattern*, whereby those who may benefit most from support are least likely to seek it. These findings resonate with theoretical notions of resource gain and loss spirals, in which resources may accumulate to promote wellbeing or deplete and reinforce strain (Bakker et al., 2023; Bakker and de Vries, 2021; Bakker and Demerouti, 2017). Although longitudinal research is needed to examine temporal processes, over time, non-disclosure may worsen health and increase risks of future presenteeism, further deteriorating health (Bergström et al., 2009; Johns, 2010).

Although no single exposure-related artifact (full-time/part-time work or months back at work) showed consistently across both occupational groups and outcomes, sensitivity analyses indicated that differences in sickness presenteeism across profiles were influenced by variation in opportunity for attendance. Sickness presenteeism may have been more uniformly distributed during the post-pandemic period, as symptom-related attendance was strongly governed by explicit rules, thereby reducing its association with psychosocial variation. In contrast, inappropriate presenteeism involves more ambiguous fitness-to-work thresholds and may be both more prevalent and less constrained by opportunity for attendance, allowing it to remain differentiated across psychosocial profiles.

An additional interpretation is that thresholds for perceived “inappropriate” may vary across psychosocial work environments. In safety-critical settings, individuals may attend duty while impaired not because they deliberately deviate from regulations, but because impairments such as fatigue or psychological distress become normalized at the level of everyday experience. Pilots sometimes attend duty despite being unfit because they misjudge their condition, and fatigue has been described as a recurrent and a normal condition in aviation (Folke and Melin, 2025). Nevertheless, the concept of normalization of mental health impairments in safety critical work has received little scholarly attention.

These findings point to a dual vulnerability in which risks to both health and safety may remain insufficiently visible, particularly in work environments characterized by less favorable psychosocial conditions. Although we cannot imply causation in any direction, it is plausible that mental health difficulties influence how individuals perceive and evaluate their psychosocial work environment. Nevertheless, as crew often refrain from reporting health concerns, dissatisfaction with the psychosocial work environment may still serve as an indirect signal of underlying distress and should not be dismissed as merely subjective. Prior research shows that strengthening psychosocial work environments reduces presenteeism (Ødegaard and Roos, 2014). Translated to aviation, addressing the psychosocial work environment has the potential to improve health and, thus, reduce the risk of pilots and cabin crew operating flights while unfit.

### 4.3 What could be done to support mental health and safety in European aviation?

As a majority of pilots and cabin crew were classified into profiles associated with elevated risk, the findings indicate a clear need for both targeted and general interventions in European aviation. While the *Strained* profile may benefit from tailored interventions addressing multiple unfavorable psychosocial conditions, improving the psychosocial conditions experienced by the most prevalent profile, the *Collegially Supported*, also represents a potential opportunity to reduce health and safety risks and disrupt patterns of mental health non-disclosure.

However, despite evidence that improving collegial relations can support wellbeing (Aust et al., 2023; Fox et al., 2022), self-reported mental health symptoms remained elevated among individuals classified into the *Collegially Supported* profile. This indicates that favorable collegial relations alone may be insufficient to mitigate health and safety risks when other psychosocial aspects of the work environment are less favorable. Similarly, greater roster influence appears to be associated with improvements in certain health and safety outcomes but does not mitigate elevated risk. Taken together, this suggests an additive pattern, in which health and safety risks reflect the accumulation of multiple unfavorable psychosocial conditions.

Although health and safety outcomes in aviation emerge from complex and interacting conditions, the present findings point to several areas that warrant attention as potential leverage points for improving crew wellbeing and fitness-to-fly behaviors. Importantly, such areas—including collegial support and the experience of roster influence—are unlikely to operate as stand-alone solutions but may nonetheless be associated with incremental improvements in both health and safety outcomes. Increased perceived roster influence—for example through participatory work design or self-scheduling (Joyce et al., 2016), or potentially in relation to part-time work, which was more common in the *Roster Empowered* profile—may support mental health, as employees with more control over their schedules are better able to accommodate personal and family needs (Aust et al., 2023; Fox et al., 2022). Mixed evidence suggests that job control can act as both a protective and a risk factor for presenteeism (Miraglia and Johns, 2016), but among pilots it appears to function primarily as a protective factor (Folke and Melin, 2022). As the *High-Flying* and the *Roster Empowered* profiles displayed lower levels of presenteeism, enhanced perceived roster influence could possibly reduce attendance in unfit states.

The results underline the importance of trustful and supportive management relations for both health (Bakker and Demerouti, 2017) and safety (Nahrgang et al., 2011), echoing European reports of a deteriorating safety climate and reduced trust in management (Folke and Melin, 2024; Jorens and Valcke, 2025). Strengthening transparent communication, trust, and mutual respect may therefore be critical for promoting wellbeing and fitness-to-fly safety behaviors.

Evidence from burnout and wellbeing research shows that interventions are most effective when organizational changes and individual support are implemented together (Aust et al., 2023; Bes et al., 2023). For aviation, this may involve examining roster influence, pay structures, and individual support systems. Minimum guaranteed pay emerged as a protective factor for profile membership in both pilots and cabin crew, but it co-occurred with other organizational characteristics that may jointly influence health and safety outcomes. As such, minimum pay may reflect broader organizational arrangements that reduce economic uncertainty but also represents one possible area for further attention.

Moreover, across both mental health and fitness-to-fly safety behaviors, cabin crew showed less favorable ratings, mirroring other results (Jorens and Valcke, 2025). While peer-support programs are mandatory for pilots in Europe, reluctance to self-disclose suggests similar mandatory support programs for cabin crew may also be warranted. Nevertheless, although such programs are generally viewed as facilitators (Minoretti, 2025), their effectiveness remains uncertain (Bråstad et al., 2024; Melin and Lång, 2023). The low willingness to disclose, even in more favorable profiles, suggests that current regulatory frameworks emphasizing individual responsibility for declaring unfitness risk overlooking the organizational pressures that can impair health and simultaneously suppress disclosure, thereby exposing a system-level contradiction that requires resolution beyond individual-level interventions (Minoretti, 2025). In line with conclusions from a recent narrative review (Minoretti, 2025), addressing this contradiction also requires attention to stigma surrounding mental health, as well as broader regulatory and systemic conditions that shape perceived career consequences, trust, and psychological safety around disclosure.

In an industry where self-disclosure cannot be assumed, psychosocial indicators may therefore be used to help detect otherwise hidden risks to occupational health and flight safety, as poorer ratings of the psychosocial work environment tend to co-occur with health impairments and presenteeism.

### 4.4 Strengths and limitations

Data collection occurred during a period of post-pandemic uncertainty, when organizational conditions were still evolving. Consequently, the extent to which the identified profiles reflect stable psychosocial characteristics versus transient responses to pandemic-related insecurity remains uncertain. Longitudinal research is therefore needed to validate these profiles and examine potential temporal changes in profile structures or membership over time, while future studies could also reduce the risk of common method bias using objective organizational measures.

Another limitation is that the latent profile models might not have fully captured within-profile variability, meaning that individual differences within each profile could be overlooked, potentially distorting the true picture of the population (Howard and Hoffman, 2018). Additionally, the semi-subjective choice of number of profiles introduces a risk of either over-extracting or under-extracting, which may lead to misinterpretation of the population structure.

One further limitation concerns the retrospective measurement of presenteeism, which—despite being adapted to the post-pandemic context—may be biased by differential opportunity for attendance; although this was addressed through sensitivity analyses in the present study, future research could examine this by more explicitly modeling time at risk. Furthermore, the use of single-item indicators introduces the possibility of measurement bias, underscoring the need for validation in future research to ensure the robustness of the identified profiles. Future studies should employ multi-item measures to more precisely measure roster influence and collegial support. Nonetheless, single items can offer reliable and valid representations of constructs and should not automatically be dismissed (Fisher et al., 2016; Matthews et al., 2022).

Completion rates also pose a limitation, as a notable dropout rate may introduce selection bias. Although modern missing data methods exist, complete case analysis was used due to concerns that imputation (by predicting missing values from observed data) may obscure heterogeneity in person-centered analyses when missingness is pronounced. At the same time, reliance on complete cases may have limited the generalizability of the results.

Whereas the surveys were broadly distributed across European aviation, the non-probability, web-based, single-mode design limits the representativeness of the samples (Cornesse and Bosnjak, 2018). Furthermore, data were collected in the ramp-up phase following the COVID-19 pandemic, a period marked by disruption and uncertainty in aviation. Consequently, caution is advised when interpreting these findings. Although representativeness cannot be guaranteed, large-scale studies in the field of aviation are rare, and this present one is the largest study on psychosocial work conditions, mental health, and safety behaviors in European aviation to date.

Another notable strength of this study is the inclusion of two occupational groups—cabin crew and pilots—who operate within similar organizational contexts in aviation. Although measurement invariance could not be established, suggesting that the psychosocial indicators may not be interpreted identically across groups, the dual-sample design allows for meaningful comparisons and broader insight into the psychosocial work environment in commercial aviation.

### 4.5 Future research

Future research could examine what drives perceptions of roster influence—whether flexibility, participatory scheduling, work hours, part-time arrangements, or other organizational factors. Moreover, although shift work and fatigue are well studied in aviation, research on aircrew’s experience of work time control appears scarce or non-existent and warrants increased attention. Future research should also complement intention-based measures by examining actual help-seeking and disclosure to employers. Moreover, further exploration is needed into the inconclusive findings on atypical employment as a potential risk factor. As the *High-Flying* profile had the highest proportion of regionally flying pilots, these workplaces could be further examined to ascertain how to organize work to increase the psychosocial work environment and health and safety practices.

While aircrew do report working while unfit, despite being legally required to refrain from duty in such states, this behavior does not appear to result in immediate safety incidents. This underscores the need for further research on how crew manage psychosocial work demands while sustaining both health and flight safety over time.

## 5. Conclusion

Four profiles of the psychosocial work environment were identified among pilots and cabin crew: *High-Flying, Roster Empowered, Collegially Supported*, and *Strained* work environments. Profiles reflecting less favorable ratings of the psychosocial work environment also displayed higher symptom levels of fatigue, depression and anxiety, as well as more presenteeism and a lower willingness to self-disclose mental health issues to the employer. These findings suggest a potential health non-disclosure pattern in aviation, where those in poorer work environments report more mental health concerns yet are less likely to self-declare them, potentially reinforcing a downward spiral in mental health and suppressed safety behaviors over time. Here, we inform future research and potential interventions by highlighting perceived roster influence, minimum guaranteed pay, and management-employee relations as psychosocial and organizational factors that warrant further attention, as they may be associated with enhancements in mental health and fitness-to-fly safety behaviors and thereby indirectly strengthen flight safety in Europe.

## Data Availability

The datasets presented in this article are not readily available because ethical permits do not allow direct sharing of these data; however, aggregated or anonymized summaries may be made available upon reasonable request. Requests to access the datasets should be directed to Filippa.folke@ki.se.
