# Supplementary material for: Pilots’ and cabin crews’ psychosocial work environment in relation to mental health and fitness-to-fly safety behaviors: latent profile analyses
Source: Front Psychol. 2026 Mar 18;17:1740908. doi: 10.3389/fpsyg.2026.1740908 (PMC13038582; doi:10.3389/fpsyg.2026.1740908)
Supplement: Supplementary file 1 [file Supplementary_File_1.pdf]

SUPPLEMENTARY MATERIAL

Cabin Crew

Indicator	Item	Response option	High-Flying	Roster Emp.	Collegially Supported	Strained
**Roster Quality** (α=.75, ω=.76, η^2=.23)			**3.84 (0.69)** a	**2.93 (0.71)** b	**2.55 (0.77)** c	**2.32 (.79)** d
Predictability	I find that I receive my roster early enough to be able to plan my life outside of work		3.80 (1.09)	2.75 (1.19)	2.50 (1.33)	2.57 (1.33)
Predictability	I find that my roster is stable enough to be able to plan my life outside of work	*(1) Strongly disagree*	3.73 (1.06)	2.95 (1.13)	2.61 (1.24)	2.40 (1.28)
Control work pace	My roster and working days are planned in such a way that I can take necessary breaks during the day (ex. going to the bathroom, having meals)	*(2) Tend to disagree* *(3) Neither* *(4) Tend to agree*	3.68 (1.12)	2.84 (1.12)	2.50 (1.16)	2.16 (1.02)
Control work pace	My roster and working days are planned in such a way that I can comply with safety policies and procedures during the day	*(5) Strongly agree*	4.23 (0.81)	3.39 (0.97)	3.03 (1.09)	2.74 (1.05)
Recovery/Predictability	My roster and work are planned in such a way that I can recover from work during my free time		3.78 (0.91)	2.75 (1.06)	2.13 (0.99)	1.78 (0.89)
**Management -Employee Relations** (α=.89, ω=.90, η^2=.45)			**4.02 (0.51)** a	**2.13 (0.61)** b	**1.97 (0.75)** c	**1.70 (0.58)** d
Staff orientation	Management show interest in the health and well-being of the staff		4.05 (0.77)	2.00 (0.93)	1.86 (1.01)	1.58 (0.94)
Staff orientation	Management acts as though they are keen to keep their staff		3.96 (0.77)	1.84 (0.94)	1.83 (1.04)	1.65 (0.97)
Management Trust	In general, employees trust management	*(1) Strongly disagree*	3.56 (0.94)	1.72 (0.75)	1.68 (0.91)	1.46 (0.70)
Appreciation	In general, employees are appreciated	*(2) Tend to disagree*	3.92 (0.77)	1.87 (0.85)	1.72 (0.93)	1.41 (0.69)
Psychological contract breach	The airline that I work for treats me according to my expectations	*(3) Neither* *(4) Tend to agree*	4.17 (0.67)	2.34 (0.93)	2.09 (0.99)	1.89 (0.99)
Psychological contract violation	I feel frustrated by how I have been treated by my airline **(R)**	*(5) Strongly agree*	1.89 (0.94)	3.72 (1.12)	3.98 (1.17)	4.13 (1.184)
Management support	If needed, my manager helps and supports me in my work		4.40 (0.61)	2.88 (1.05)	2.64 (1.15)	2.09 (1.05)
**Safety Climate** (α=.78, ω=.79, η^2=.11)			**4.44 (0.54)** a	**3.94 (0.67)** b	**3.71 (0.82)** c	**3.33 (0.84)** d
Safety commitment	Safety is taken seriously in this company and there is a strong safety culture in place (incl. from top management)	*(1) Strongly disagree*	4.68 (0.57)	4.12 (0.91)	3.93 (1.09)	3.54 (1.15)
Feedback	We get timely feedback on the safety issues we raise	*(2) Tend to disagree*	4.10 (0.92)	3.44 (1.05)	3.16 (1.17)	2.82 (1.12)
Learning	We learn lessons from safety-related incidents or occurrence investigations	*(3) Neither* *(4) Tend to agree*	4.60 (0.62)	4.39 (0.74)	4.24 (0.89)	3.89 (0.92)
Communication	There is good communication up and down the organization about safety	*(5) Strongly agree*	4.41 (0.69)	3.83 (0.92)	3.52 (1.10)	3.08 (1.06)
**Job Insecurity** (α=.82, α=.82, η^2=.06)			**2.81 (1.12)** a	**3.53 (1.05)** b	**3.75 (1.10)** c	**3.76 (1.10)** bc
Job loss	I worry about losing my job	*As above*	2.57 (1.22)	3.21 (1.19)	3.44 (1.27)	3.54 (1.26)

Employment stability	I worry about the future stability of my employment		3.04 (1.30)	3.86 (1.11)	4.07 (1.14)	3.98 (1.15)
**Roster Influence** (η²=.78)	I feel that I can influence my roster	*As above*	**3.80 (0.75)** $_a$	**3.58 (0.59)** $_b$	**1.47 (.49)** $_c$	**1.42 (0.56)** $_c$
**Collegial Support** (η²=.41)	If needed, my colleagues help and support me in my work	*As above*	**4.65 (0.53)** $_a$	**4.37 (0.67)** $_b$	**4.52 (0.50)** $_c$	**2.75 (0.45)** $_d$

Note. Subscripts ($_{a, b, c, d}$) denote significant pairwise differences between profiles ($p < .05$). α = Cronbach's alpha, ω = McDonald's omega (internal consistency of indicators), η² = eta squared (effect size, proportion of variance explained by profile membership)

Pilots

Indicator	Item	Response option	High-Flying	Roster Emp.	Collegially Supported	Strained
**Roster Quality** (α=.80, ω=.81, η²=.31)			**3.84 (0.74)** $_a$	**3.29 (0.74)** $_b$	**2.70 (0.82)** $_c$	**2.32 (.79)** $_c$
Predictability	I find that I receive my roster early enough to be able to plan my life outside of work		3.53 (1.21)	2.74 (1.35)	2.39 (1.32)	2.64 (1.31)
Predictability	I find that my roster is stable enough to be able to plan my life outside of work	*(1) Strongly disagree*	3.89 (1.13)	3.39 (1.16)	2.62 (1.31)	2.74 (1.29)
Control work pace	My roster and working days are planned in such a way that I can take necessary breaks during the day (ex. going to the bathroom, having meals)	*(2) Tend to disagree* *(3) Neither* *(4) Tend to agree*	3.82 (1.11)	3.26 (1.18)	2.70 (1.22)	2.64 (1.15)
Control work pace	My roster and working days are planned in such a way that I can comply with safety policies and procedures during the day	*(5) Strongly agree*	4.07 (0.89)	3.66 (0.98)	3.13 (1.09)	3.00 (1.09)
Recovery/Predictability	My roster and work are planned in such a way that I can recover from work during my free time		3.88 (0.95)	3.42 (0.98)	2.67 (1.10)	2.67 (1.13)
**Management -Employee Relations** (α=.94, ω=.94, η²=.77)			**3.81 (0.59)** $_a$	**2.02 (0.52)** $_b$	**1.79 (0.56)** $_c$	**1.99 (0.63)** $_b$
Staff orientation	Management show interest in the health and well-being of the staff		3.51 (0.86)	1.74 (0.75)	1.47 (.67)	1.67 (0.79)
Staff orientation	Management acts as though they are keen to keep their staff		3.65 (0.92)	1.70 (0.86)	1.50 (.76)	1.86 (0.94)
Management Trust	In general, employees trust management	*(1) Strongly disagree*	3.36 (0.94)	1.44 (0.59)	1.36 (.59)	1.65 (0.78)
Appreciation	In general, employees are appreciated	*(2) Tend to disagree*	3.87 (0.79)	1.91 (0.86)	1.60 (0.78)	1.89 (0.89)
Psychological contract breach	The airline that I work for treats me according to my expectations	*(3) Neither* *(4) Tend to agree*	3.98 (0.72)	2.22 (0.92)	1.97 (0.93)	2.17 (0.95)
Psychological contract violation	I feel frustrated by how I have been treated by my airline **(R)**	*(5) Strongly agree*	1.66 (0.82)	3.67 (1.10)	3.89 (1.15)	3.61 (1.18)
Management support	If needed, my manager helps and supports me in my work		4.40 (0.61)	2.85 (.97)	2.58 (1.06)	2.34 (.89)
**Safety Climate** (α=.88, ω=.89, η²=.24)			**4.11 (0.72)** $_a$	**3.78 (0.78)** $_b$	**3.19 (0.90)** $_c$	**2.91 (0.92)** $_d$
Safety commitment	Safety is taken seriously in this company and there is a strong safety culture in place (incl. from top management)	*(1) Strongly disagree* *(2) Tend to disagree*	4.35 (0.80)	3.89 (0.93)	3.29 (1.14)	3.11 (1.09)

Feedback	We get timely feedback on the safety issues we raise	*(3) Neither*	3.92 (0.90)	3.52 (0.97)	3.02 (1.07)	2.72 (1.11)
Learning	We learn lessons from safety-related incidents or occurrence investigations	*(4) Tend to agree* *(5) Strongly agree*	4.19 (0.82)	4.13 (0.84)	3.63 (1.00)	3.22 (1.10)
Communication	There is good communication up and down the organization about safety		3.98 (0.87)	3.58 (0.96)	2.84 (1.15)	2.60 (1.10)
**Job Insecurity** (α=.84, ω=.84, η^2=.12)			**2.72 (1.08)** $_a$	**3.13 (1.04)** $_b$	**3.56 (1.08)** $_c$	**3.55 (.95)** $_c$
Job loss	I worry about losing my job	*As above*	2.51 (1.17)	2.75 (1.14)	3.25 (1.22)	3.31 (1.08)
Employment stability	I worry about the future stability of my employment		2.94 (1.19)	3.51 (1.17)	3.88 (1.14)	3.78 (1.01)
**Roster Influence** (η^2=.49)	I feel that I can influence my roster	*As above*	**3.20 (1.15)** $_a$	**3.93 (0.51)** $_b$	**1.61 (.69)** $_c$	**1.78 (0.88)** $_d$
**Collegial Support** (η^2=.42)	If needed, my colleagues help and support me in my work	*As above*	**4.40 (0.56)** $_a$	**4.36 (0.56)** $_a$	**4.37 (0.53)** $_a$	**2.71 (0.50)** $_b$

Note. Subscripts denote significant pairwise differences between profiles ($p < .05$). α = Cronbach's alpha, ω = McDonald's omega (internal consistency of indicators), η^2 = eta squared (effect size, proportion of variance explained by profile membership)
