# Supplementary material for: Pilots’ and cabin crews’ psychosocial work environment in relation to mental health and fitness-to-fly safety behaviors: latent profile analyses
Source: Front Psychol. 2026 Mar 18;17:1740908. doi: 10.3389/fpsyg.2026.1740908 (PMC13038582; doi:10.3389/fpsyg.2026.1740908)
Supplement: Supplementary file 2 [file Supplementary_file_2.docx]

APPENDIX A

**Completion rate analyses**

Demographic questions such as gender were located at the end of the surveys; consequently, non-response at later stages created missing demographic information. However, organizational variables were collected earlier in the survey, allowing a completion rate analysis based on gender non-response across organizational characteristics.

For pilots, results showed no significant differences in completion by type of service provided by the airline. However, differences emerged for type of employment, χ²(1, N=6,141)=8.79, p=.003, with typically employed pilots (72.7%) being more likely to complete the full survey compared to those with other types of employment (66.6%). Significant differences were also observed by type of operation, χ²(1, N=6,141)=16.59, p=.002, where long-haul pilots had the highest completion rate (75.4%), followed by short-haul (71.7%), regional (69.1%), other (68.8%), and mixed (68.6%).

For cabin crew, results showed no significant differences in completion by type of employment, or type of operation. However, significant differences were found for type of service provided by the airline, χ²(3, N=2,741)=13.36, p=.004. Cabin crew working within business aviation services were notably less likely to complete the full survey (41.2%) compared to those in scheduled passenger services (60.7%), non-scheduled charter (57.9%), or other services (59.3%).

These results indicate that completion rates were somewhat related to employment context, though the overall impact on the representativeness of the main pilot and cabin crew groups is likely limited.

APPENDIX B

| *Measurement interpretations, implications, and internal consistency of psychosocial indicators (cabin crew & pilots)* | | | | | |
| --- | --- | --- | --- | --- | --- |
| **INDICATOR (No of items)** | **Measurement Interpretation** | **Implications of high values** |  | α/ω | |
|  |  |  |  | Cabin Crew | Pilots |
| *Roster Quality (5)* | Measures roster stability, predictability, intensity, and opportunities for breaks and recovery. | Crew members perceive their roster as offering predictability, control, and rest opportunities, enabling them to manage their time and activities within their work schedule effectively. |  | .75/.76 | .80/.81 |
| *Management-Employee Relations (7)* | Measures the employee’s experiences of trust, support, staff orientation, and appreciation by their management, alongside cognitive and emotional aspects such as frustration with treatment by the airline. | Crew experience positive management-employee relations characterized by trust, support, and appreciation. |  | .89/.90 | .94/.94 |
| *Safety Climate (4)* | Measures the presence of a robust safety culture, the effectiveness of incident learning processes, and the quality of safety communication within the organization. | A robust safety culture marked by commitment, effective communication and learning from mistakes. |  | .78/.79 | .88/.89 |
| *Job Insecurity (2)* | Measures worry regarding potential job loss and uncertainties regarding future job stability. | Elevated levels of concern regarding job security. |  | .82/.82 | .84/.84 |
| *Collegial Support (1)* | Measures the perception of collegial support in times of need. | The presence of strong collegial support. |  | - | - |
| *Roster Influence (1)* | Measures the perception of roster influence. | The employee feels they have a significant level of influence over their work schedules. |  | - | - |

APPENDIX C

| *Frequency distribution of depressive and anxiety symptom levels (HADS) across psychosocial profiles among cabin crew and pilots* | | | | | |
| --- | --- | --- | --- | --- | --- |
|  |  | **High-Flying** | **Roster Emp.** | **Collegially Supported** | **Strained** |
| **Cabin Crew** | **Depressive Symptoms (HADS-D)** |  |  |  |  |
|  | <8 (No symptoms) | 95.2% | 74.8% | 65.3% | 49.6% |
|  | 8-10 (Mild symptoms) | 4.2% | 16.7% | 21.8% | 31.2% |
|  | 11-14 (Moderate symptoms) | 0% | 7.5% | 9.8% | 14.4% |
|  | >15 (Severe symptoms) | 0.6% | 0.9% | 3.2% | 4.8% |
|  |  |  |  |  |  |
|  | **Anxiety Symptoms (HADS-A)** |  |  |  |  |
|  | <8 (No symptoms) | 80.0% | 57.9% | 46.2% | 34.7% |
|  | 8-10 (Mild symptoms) | 12.1% | 22.0% | 23.4% | 26.6% |
|  | 11-14 (Moderate symptoms) | 7.3% | 16.7% | 21.2% | 29.0% |
|  | >15 (Severe symptoms) | 0.6% | 3.4% | 9.3% | 9.7% |
|  |  |  |  |  |  |
| **Pilots** | **Depressive Symptoms (HADS-D)** |  |  |  |  |
|  | <8 (No symptoms) | 92.3% | 84.3% | 75.4% | 63.3% |
|  | 8-10 (Mild symptoms) | 6.1% | 11.4% | 16.4% | 23.1% |
|  | 11-14 (Moderate symptoms) | 1.5% | 4.0% | 6.8% | 10.4% |
|  | >15 (Severe symptoms) | 0.1% | 0.2% | 1.4% | 3.2% |
|  |  |  |  |  |  |
|  | **Anxiety Symptoms (HADS-A)** |  |  |  |  |
|  | <8 (No symptoms) | 87.7% | 78.1% | 69.5% | 59.5% |
|  | 8-10 (Mild symptoms) | 9.2% | 15.6% | 18.8% | 23.3% |
|  | 11-14 (Moderate symptoms) | 2.7% | 5.9% | 9.3% | 12.1% |
|  | >15 (Severe symptoms) | 0.4% | 0.5% | 2.4% | 5.1% |
|  |  |  |  |  |  |
| *Note.* The table presents the percentage of participants falling into each symptom severity category − none (<8), mild (8-10), moderate (11-14), and severe (>15) − based on the Hospital Anxiety and Depression Scale (HADS). Frequencies are shown separately for depressive symptoms (HADS-D) and anxiety symptoms (HADS-A) and stratified by occupational group (cabin crew and pilots) and latent psychosocial work profile (High-Flying, Roster Empowered, Collegially Supported, and Strained). | | | | | |
